# Disruption of the dividing membrane in monochorionic diamniotic twin pregnancy

**DOI:** 10.1002/ccr3.2570

**Published:** 2019-11-28

**Authors:** Tatsunori Shiraishi, Yoshie Shibata, Momoko Owada, Shunji Suzuki

**Affiliations:** ^1^ Deaprtment of Obstetrics and Gynecology Japanese Red Cross Katsushika Maternity Hospital Tokyo Japan

**Keywords:** disruption of dividing membrane, fetoscopic laser photocoagulation, monochorionic diamniotic twin pregnancy, ultrasonography

## Abstract

Disruption of the dividing membrane in monochorionic diamniotic twins is associated with a higher incidence of prematurity and neonatal morbidity. In the present case, a cramped fragment of the dividing membrane could be noted following fetoscopic laser photocoagulation.

## Graphical abstract

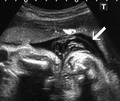

A diagnosis of twin‐twin transfusion (TTTS) stage II was made at 18 weeks of gestation, and fetoscopic laser photocoagulation (FLP) of placental communicating vessels was performed. In our institute, uncomplicated monochorionic diamniotic (MD) twin pregnancies are managed by ultrasonography interval of 1‐2 weeks; however, if some complications can be recognized, the management is performed at 3‐ to 7‐day intervals. At 28 weeks of gestation, the dividing membrane could not be demonstrated by ultrasonography, and the presence of a cramped fragment of the membrane was suspected near one fetus (Figure 1). Elective cesarean section was performed at 31 weeks of gestation, and healthy male infants with loosely entangled umbilical cords were delivered from a single gestational sac. Macroscopic examination showed that the placenta had a falciform remnant of the disrupted dividing membrane (Figures 2 and 3).

**Figure 1:**
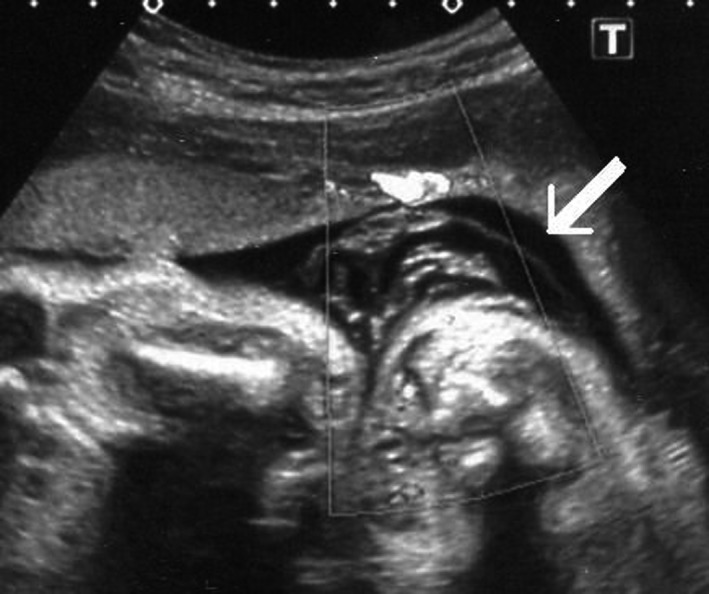
The presence of a cramped fragment of the dividing membrane near one fetus suspected at 28 wk of gestation (white arrow)

**Figure 2:**
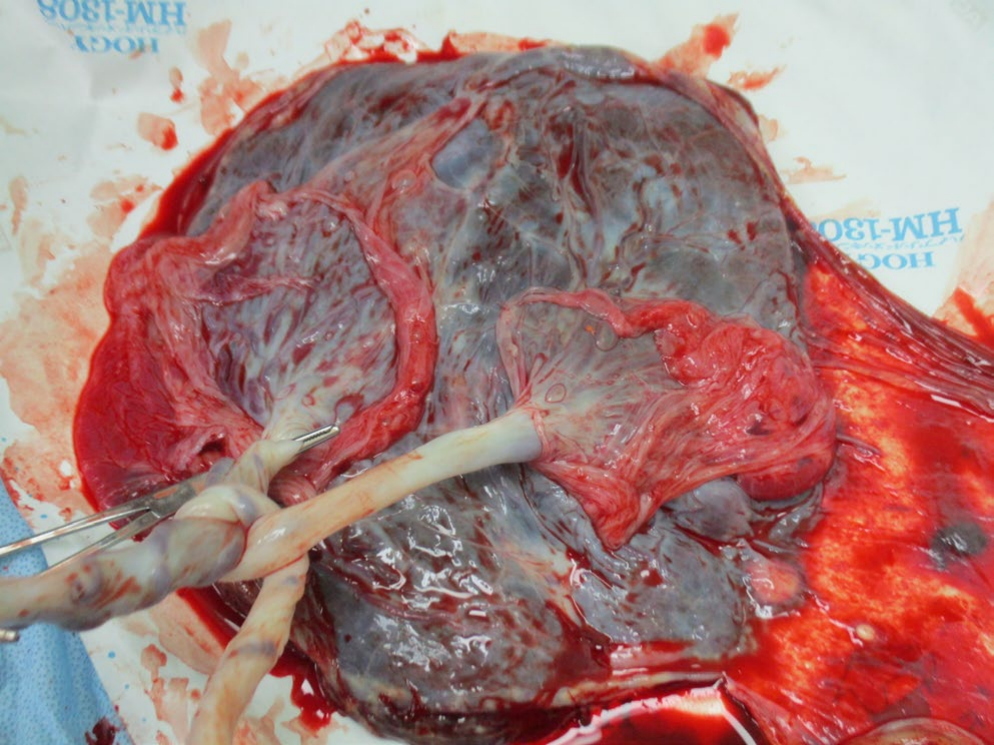
Cord entanglement in the placenta

**Figure 3:**
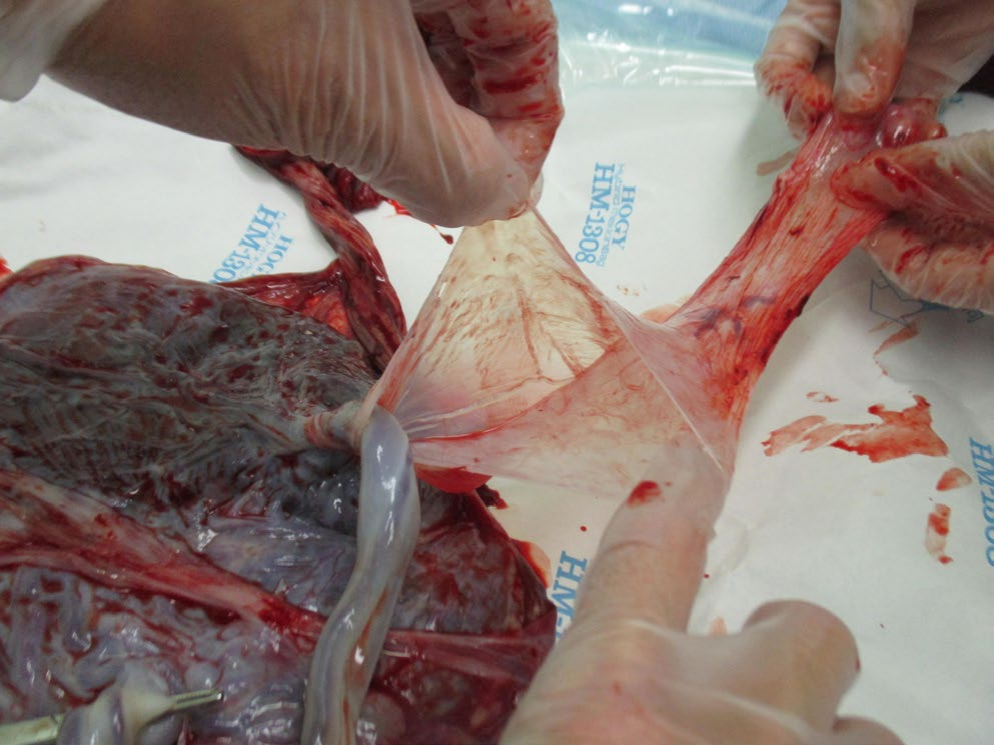
Falciform remnant of the disrupted dividing membrane in the placenta

Iatrogenic disruption of the dividing membrane by laser therapy has been reported to be associated with a lower gestational age at delivery and increased neonatal morbidity.^1^, ^2^ Therefore, it is very important to actively search for signs of disruption; however, an ultrasonographic search for disruption may be difficult.^1^, ^2^ In the present case, it was fortunate that we were able to confirm a falciform remnant of the disrupted dividing membrane.

## CONFLICT OF INTEREST

None declared.

## AUTHORS' CONTRIBUTIONS

TS (Primary author and outpatient physician): analyzed the data from the ultrasonographic examination, and wrote and revised the manuscript; YS and MO (Primary care physicians in hospital): performed cesarean section and examined the placenta; SS (Director of the Department): involved in the conception of the study, analyzed the data comprehensively, and drafted and revised the manuscript.
